# Supplementary material for: Cytoglobin regulates NO-dependent cilia motility and organ laterality during development
Source: Nat Commun. 2023 Dec 14;14:8333. doi: 10.1038/s41467-023-43544-0 (PMC10721929; doi:10.1038/s41467-023-43544-0)
Supplement: Supplementary file 1 — Supplementary Information [file 41467_2023_43544_MOESM1_ESM.pdf]

SUPPLEMENTARY INFORMATION

SUPPLEMENTARY FIGURES

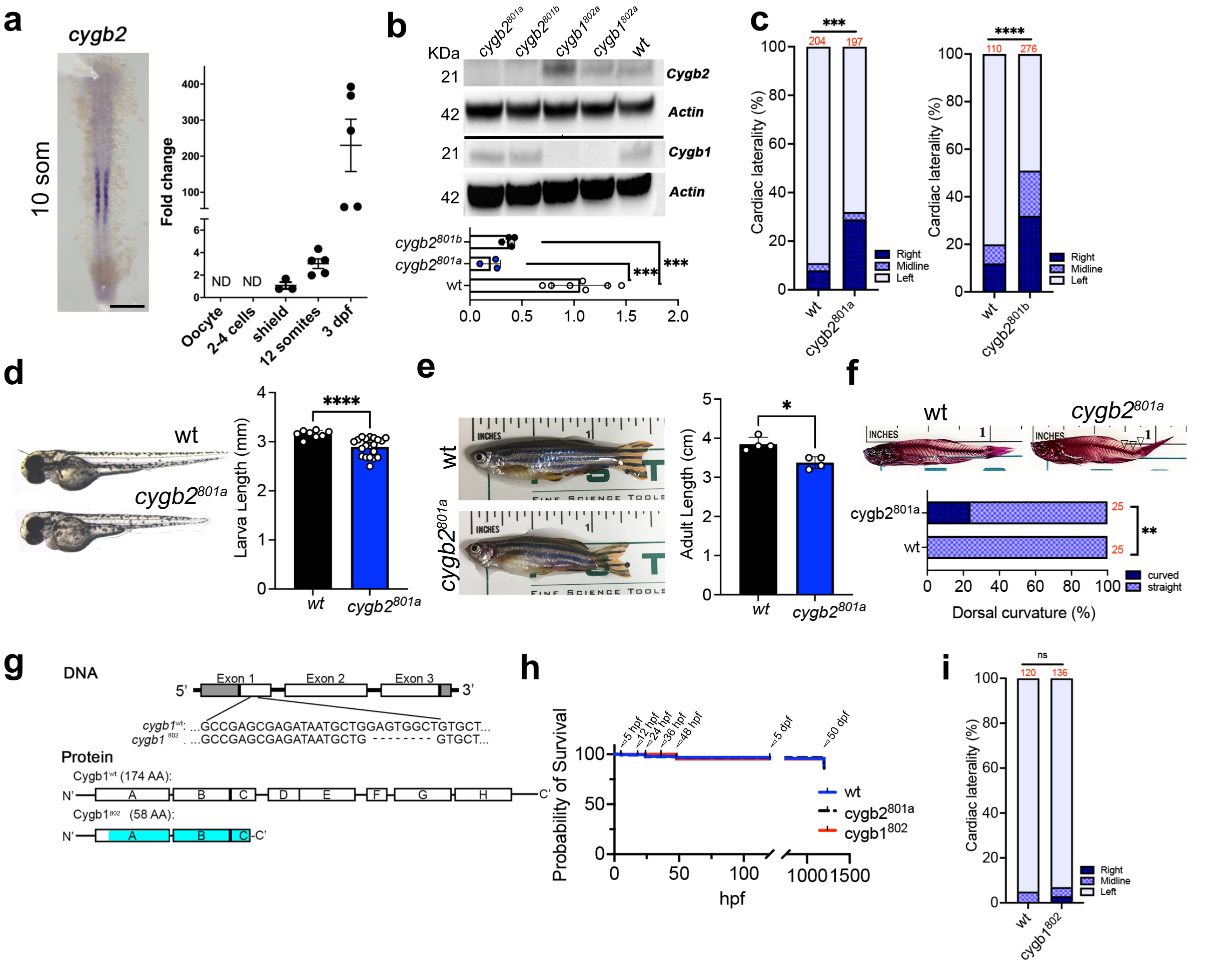

Supplemental Figure 1. (a) Left: *cygb2* in situ hybridization in wt embryos at 9-10 somite (dorsal view, scale bar = 10 μm). Right: qRT-PCR of *cygb2* transcript relative to *β-actin* showing detectable levels starting at shield stage. N=3-5 biological replicates, each sample representing 50 pooled embryos. **(b)** Representative Western blots for Cygb2 and Cygb1 relative to beta-actin of adult zebrafish whole brain lysate collected from *cygb2*^{801a}, *cygb2*^{801b}, *cygb1*^{802a} mutants and wt. No bands corresponding to Cygb2 (21 kDa) are detectable in the *cygb2*^{801a} nor in the

cygb2^{801b} mutants. No bands corresponding to Cygb1 (21 kDa) are detectable in the *cygb1*^{802a} mutants. Below, quantification of Cygb2 relative to beta-actin. Means are ± SD. Student's t-test, two-tailed, *** P< 0.001, **** P< 0.0001. **(c)** Percentage of embryos with left, midline or right sided hearts in wt and *cygb2*^{pt801a} and *cygb2*^{pt801b}. Number of embryos analyzed is shown above the graph in red. The Chi-squared test was used to determine statistical significance, *** P< 0.001, **** P< 0.0001. **(d)** Wt and *cygb2*^{pt801a} larva at 3 dpf, comparing body axis length. Means are ± SD. Student's t-test, two-tailed, *** P< 0.001. **(e)** Wt and *cygb2*^{pt801a} adult fish comparing body axis length. Means are ± SD. Student's t-test, two-tailed, * P< 0.05. **(f)** Wt and *cygb2*^{pt801a} adult fish comparing spinal cord curvature indicated by arrowheads and quantified in the graph. Number of embryos analyzed is reported in red in the graph. The Chi-squared test was used to determine statistical significance, ** P <0.01. **(g)** CRISPR/Cas9 mediated genome editing of *cygb1*. gRNA was targeted to exon 1 and resulted in 8 bp frame shift mutations (beginning in the blue shaded region of the predicted protein structure) named *cygb1*^{802a}. The eight globin protein helices (labeled A-H) are represented by boxes, with out-of-frame amino acids shaded blue. **(h)** Kaplan-Meier curve showing survival probabilities in wt, *cygb2*^{pt801a}, *cygb1*^{pt802a} between 5 hours post fertilization (hpf) and 50 days post fertilization (dpf). **(i)** Percentage of embryos with left, midline or right sided hearts in wt and *cygb1*^{pt802a}. Number of embryos analyzed is shown above the graph in red. The Chi-squared test was used to determine statistical significance, ns = not significant. Source data are provided as source data file.

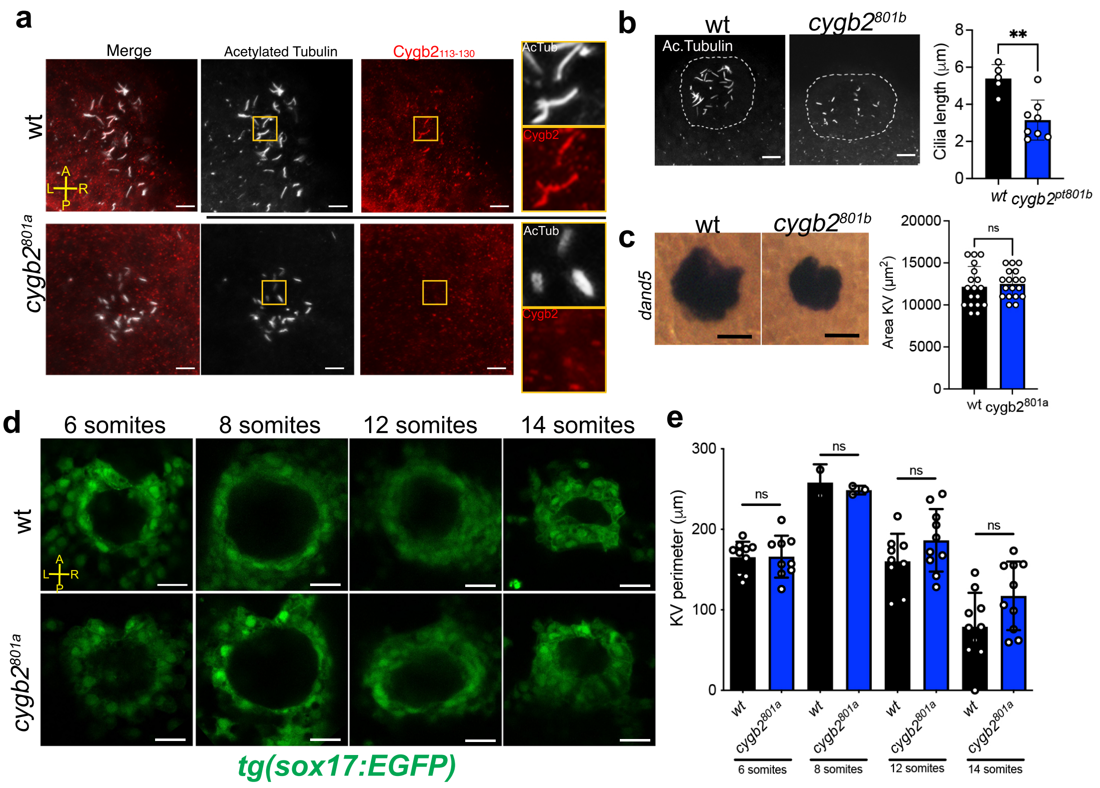

Supplemental Figure 2. (a) KV of wt and *cygb2*^{pt801a} embryos at 10 somite stained with anti-acetylated tubulin (white) to visualize cilia and a recombinant peptide anti-Cygb2$_{113-130}$ antibody (red) on whole mount embryos. The squared images outlined yellow are enlargement of selected areas in the figure as indicated. A- anterior, P- posterior, L- left, R- right. Scale bar = 10 µm. **(b)** On the left, immunostaining of acetylated tubulin (white) in wt and *cygb2*^{pt801b} embryos at 10 som. The KV is outlined with a white dashed line. Scale bar = 10 µm. On the right, quantification of cilia length in wt and *cygb2*^{pt801b} embryos. Means are ± SD (n=4-8 embryos). Student's t-test, two-tailed, ** P<0.01. **(c)** *dand5* expression labeling the KV by whole mount in situ hybridization. Scale bar = 100 µm. On the left, quantification of the KV area in wt and *cygb2*^{pt801a}. Means are ± SD (n=20 embryos). Student's t-test, two-tailed, ns = not significant. Image orientation is indicated: A- anterior, P- posterior, L- left, R- right. **(d)** Confocal images of

cygb2^{801a}*;tg(sox17:EGFP)* compared to *tg(sox17:EGFP)* at different stages of development. Images are 3D confocal projections. Scale bar = 10 µm. **(e)** Quantification of KV size by measuring the circumference of largest KV section in three central confocal Z planes. Comparisons were made between wt and *cygb2*^{pt801a} at each time point. Student's t-test, two-tailed, ns = not significant. Source data are provided as source data file.

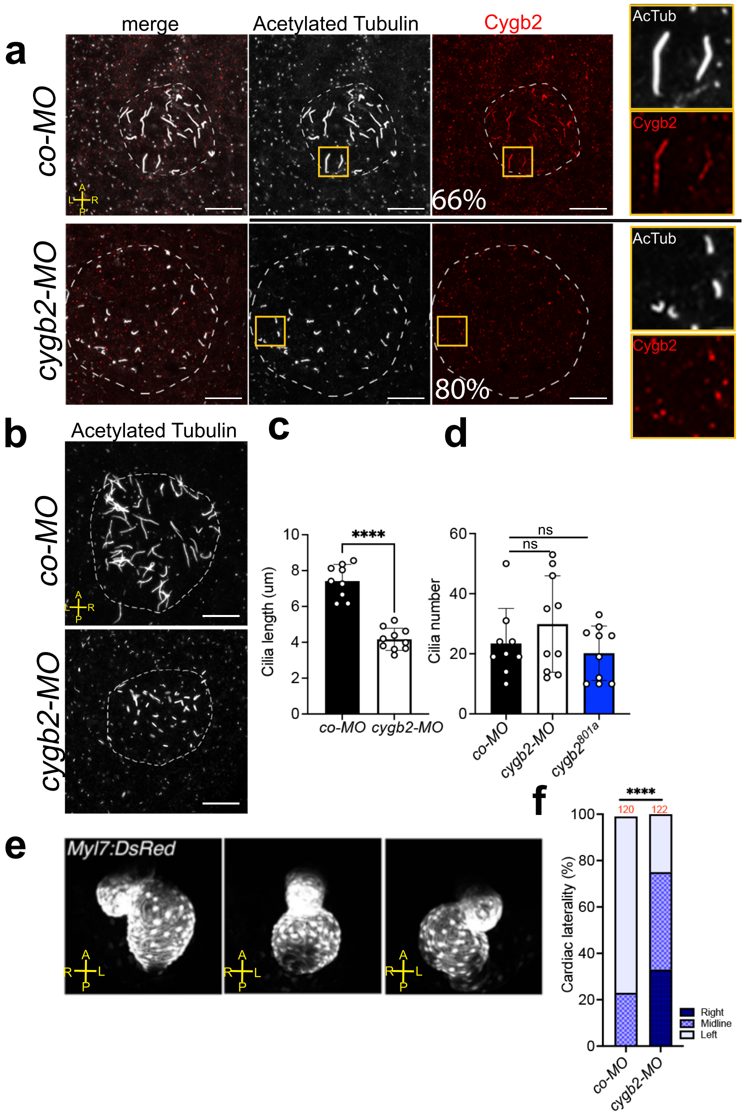

Supplemental Figure 3. (a) Immunostaining of acetylated tubulin (white) and Cygb2 (red) in control morpholino (co-MO) and *cygb2* morpholino (*cygb2*-MO) injected embryos. Images are representative of the indicated percentages. The KV is outlined with a white dashed line and shows decreased Cygb2 staining in *cygb2* morphants. The squared images outlined yellow are enlargement of selected areas in the figure as indicated. Scale bar = 10 µm. **(b)** KV cilia of co-MO and *cygb2*-MO injected embryos. Cilia are immune-stained for acetylated tubulin. Scale bar = 10 µm. **(c)** Average cilia length, means are ± SD (n=9-10 embryos). Student's t-test, two-tailed, **** P <0.0001**.** **(d)** Quantification of KV cilia number in *cygb2* morphants and $cygb2^{801a}$ mutants, means are ± SD (n=8-10 embryos). Student's t-test, two-tailed, ns = not significant. **(e)** Representative confocal 3D projections of *tg(myl7:DsRed*) hearts at 2 dpf injected with co-MO and *cygb2*-MO. **(f)** Quantification of left, midline and right sided hearts in control and *cygb2* morphants. Number of embryos analyzed is shown in red above the graph. The Chi-squared test was used to determine statistical significance. Image orientation is indicated: A- anterior, P-posterior, L- left, R- right. Source data are provided as source data file.

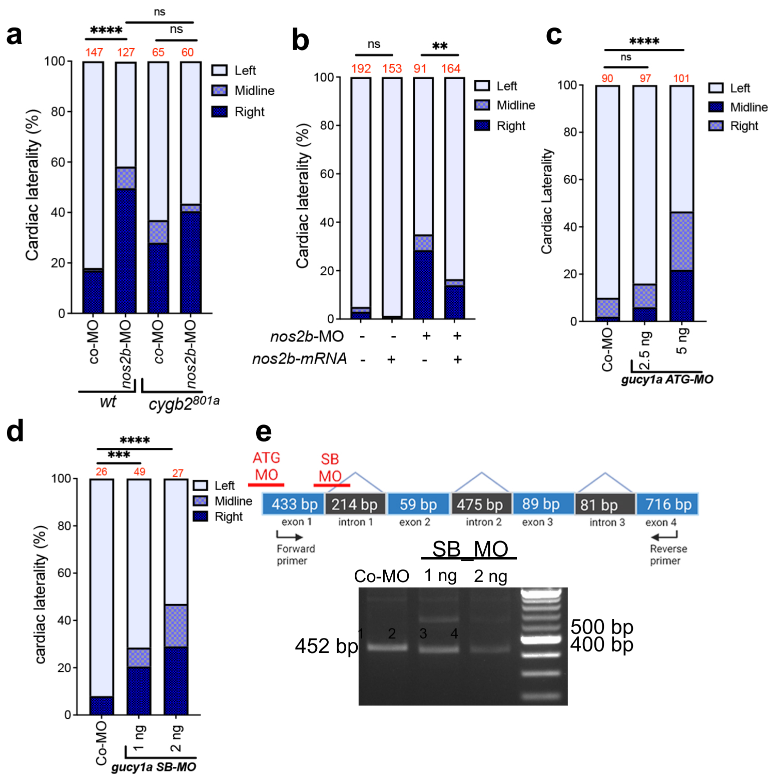

Supplemental Figure 4. (a) Percentage of embryos with left, midline or right cardiac position following injection of control (co) or *nos2b* morpholino (MO) in wt or *cygb2*^{pt801a} embryos. **(b)** Percentage of wt embryos with left, midline or right cardiac position following injection of *nos2b* MO with or without co-injection of a *nos2b* mRNA construct. **(c-d)** Percentage of wt embryos with left, midline or right cardiac position following injection of control or *gucy1a1* ATG-MO or *gucy1a1* splice-blocking (SB) MO. The number of embryos analyzed is above the graph in red text. The Chi-squared test was used to determine statistical significance. **(e)** Top, schematic of the *gucy1a* gene notating the morpholino binding sites. Bottom, RT-PCR demonstrating changes in transcript in control, 1 ng, and 2 ng *gucy1a* SB-MO.

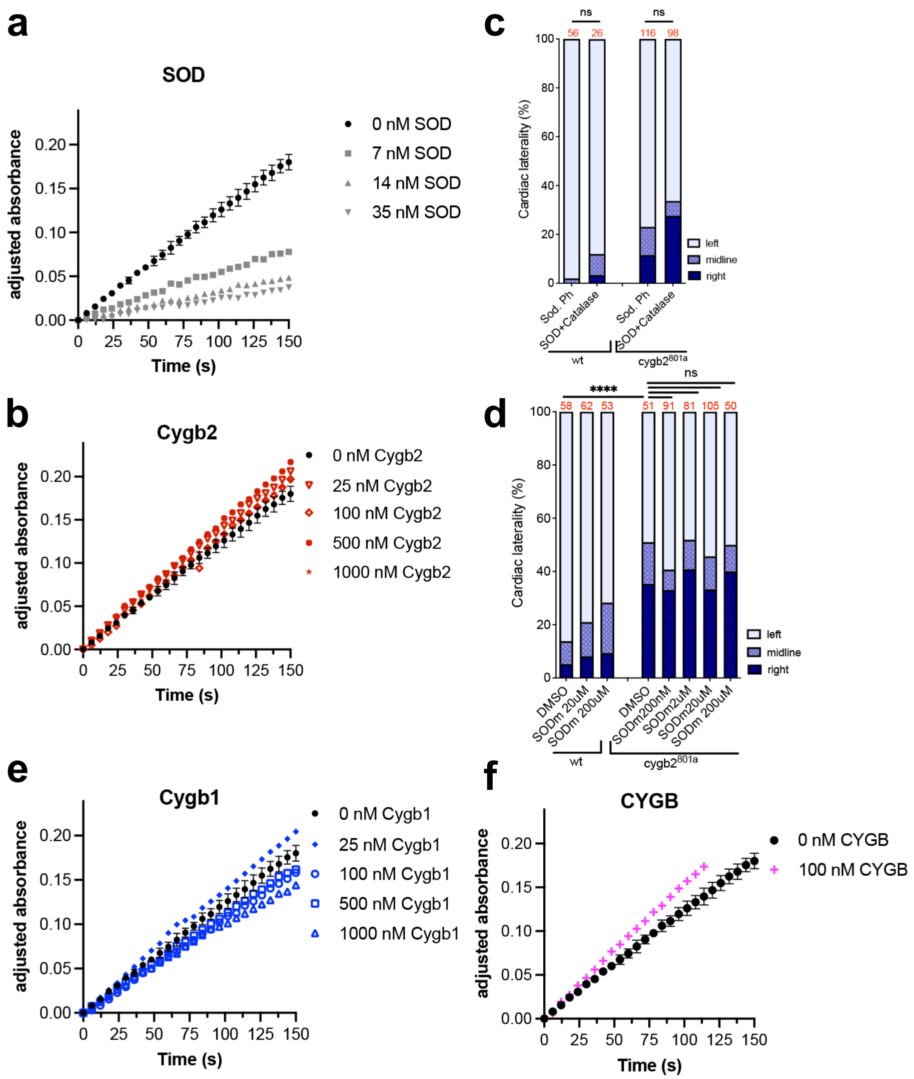

Supplemental Figure 5. (a) Measurement of the SOD activity of native SOD and (**b**) Cygb2 at different concentrations assayed by ferricytochrome *c* reduction. Adjusted absorbance at 550 nm, denoting the buildup of ferrocytochrome *c*, is shown. **(c)** Percentage of embryos with left, midline or right cardiac looping following treatment with a SOD/Catalase in sodium phosphate. **(d)** Percentage of embryos with left, midline or right cardiac looping following treatment with SOD mimic (SODm) in DMSO at different concentrations. Number of embryos analyzed is represented above the graph in red text. The Chi-squared test was used to determine statistical

significance. **(e)** Measurement of the SOD activity of Cygb1 and human CYGB (**f**) at different concentrations assayed by ferricytochrome *c* reduction.

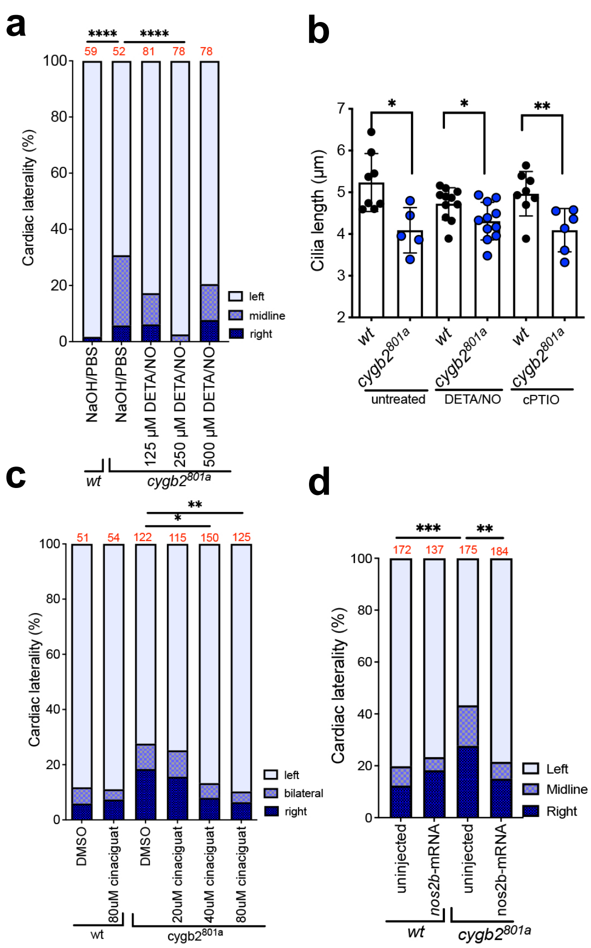

Supplemental Figure 6. (a) Percentage of embryos with left, midline or right cardiac position following treatment with a DETA/NO dose response in wt and *cygb2^{pt801a}* embryos (doses indicated in the graph). The Chi-squared test was used to determine statistical significance. **(b)** Average cilia length in wt and *cygb2^{pt801a}* embryos treated with 250 DETA/NO µM or 500 µM cPTIO. Means are ± SD. Student's t-test, two-tailed, * P <0.05, ** P <0.01. **(c)** Percentage of embryos with left, midline or right cardiac position following treatment with different doses of

cinaciguat (doses indicated in the graph). **(d)** Percentage of embryos with left, midline or right cardiac position following injection with the *nos2b*-mRNA construct in wt or *cygb2*^{pt801a} embryos. Number of embryos analyzed is represented above the graph in red text. The Chi-squared test was used to determine statistical significance. Source data are provided as source data file.

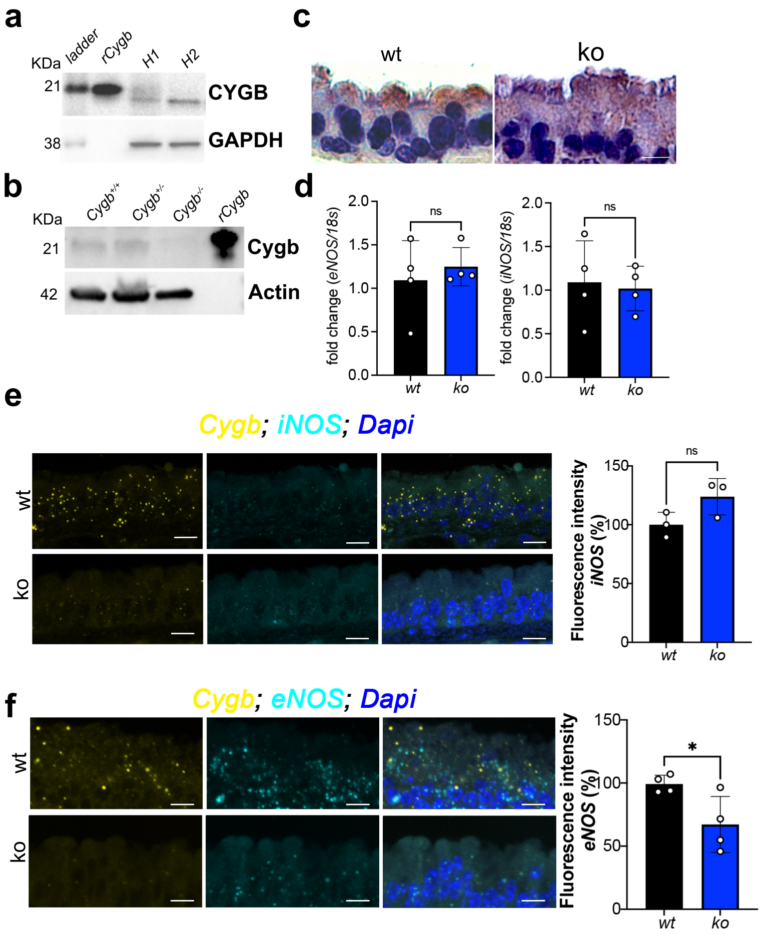

Supplemental Figure 7. (**a**) Representative Western blots for human CYGB relative to GAPDH of human trachea scrapes collected from two healthy subjects (H1 and H2). A band corresponding to CYGB (~21 kDa) is detectable in both human samples. **(b)** Representative Western blots for mouse Cygb relative to actin of mouse trachea lysate collected from *Cygb*$^{-/-}$,

Cygb$^{-/+}$, *Cygb*$^{+/+}$. **(c)** Immunostaining of Cygb in mouse tracheas visualized by DAB staining. **(d)** Quantification of *iNOS* transcript by qRT-PCR. **(e)** RNA scope on ciliated epithelium from mouse trachea showing *Cygb* (yellow) and *iNOS* (cyan) expressed in the same cells. **(f)** RNA scope on ciliated epithelium from mouse trachea showing *Cygb* (yellow) and *eNOS* (cyan) expressed in the same cells. On the right, quantification of fluorescence intensity measured on RNAscope images in E-F. Means are ± SD. Student's t-test, two-tailed, * P <0.05, ns = not significant. Source data are provided as source data file.

Table S1: Primers and oligos used in this study.

Primer Name	F/R	Sequence (5' – 3')	Assay
cygb2 gRNA_801a	F	ATTTAGGTGACACTATAGGTGGAGCGGGGCATCATTAGTTTTAGAGCTAGAAATAGC	gRNA PCR
cygb2 gRNA_801b	F	ATTTAGGTGACACTATAGGGCATCATTAAGGACACCTGTTTTAGAGCTAGAAATAGC	gRNA PCR
cygb1 gRNA_802	F	ATTTAGGTGACACTATAGGAGCGAGATAATGCTGGAGGTTTTAGAGCTAGAAATAGC	gRNA PCR
gRNA universal primer	R	AAAAGCACCGACTCGGTGCCACTTTTTCAAGTTGATAACGGACTAGCCTTATTTTAACTTGCTATTTCTAGCTCTAAAAC	gRNA PCR
cygb2 _Ex1	F	CACAGCCTGTCATTTCACCA	Genotyping
cygb2 _Ex1	R	ATGATGGATAAATTTGCACACTG	Genotyping
cygb1 _Ex1	F	GCGAGCGAGAGCCTCATAAAGGGCTG	Genotyping
cygb1 _Ex1	R	GCCAGCAGTAGTAAATGATTGCTGTGCGCG	Genotyping
cygb2 AUG-MO		CATCCTCCCTCTCTTTCTCCATGCC	MO knockdown
gucy1a1 AUG-MO		GCGTAATGGTCCTCTCCAAACACAC	MO knockdown
gucy1a1 SB-MO		ACATCGGACAAACGATTTTACCTCT	MO knockdown

nosb2 AUG-MO		CCTGGTTGCCCATGTGTTTCTGAAA	MO knockdown
control-MO		CCTCTTACCTCAGTTACAATTTATA	MO knockdown
*gucy1a1*_ex1-4	F	GGAGAGGACCATTACGCAAA	MO RT-PCR
*gucy1a1*_ex1-4	R	GGAGAGGACCATTACGCAAA	MO RT-PCR
cygb2 -Cla1	F	TATAATCGATGCCACCATGCACACTCTCTACTCTCT	*cygb2* expression construct
cygb2 -Xho1	R	TATACTCGAGGACTGCTGAGCT GGATAGTT	*cygb2* expression construct
cygb2 -rProbe	F	CACAGCCTCTCATTTCACCA	ISH
cygb2 -rProbe	R	TAATACGACTCACTATAGGGAGA CGTGGCATAGACAACATCTGA	ISH
spaw -rProbe	F	GTCCTGAGCTTGATTGCACA	ISH
spaw -rProbe	R	TAATACGACTCACTATAGGGAGACTTTCCACACGGTTTCGTTT	ISH
lft2 -rProbe	F	AGAAGCGGGATTTGGAGAAT	ISH
lft2 -rProbe	R	TAATACGACTCACTATAGGGAGAGCAGAGGTGCTTTCTTTTGG	ISH
myl7 -rProbe	F	GACCAACAGCAAAGCAGACA	ISH

myl7 -rProbe	R	TAATACGACTCACTATAGGGAGAGGGTCATT AGCAGCCTCTTG	ISH
foxa3 -rProbe	F	CTTCAACGATTGCTTCGTCA	ISH
foxa3 -rProbe	R	TAATACGACTCACTATAGGGAGAGGGTCTGG TAGTAGGCACCA	ISH
