# Supplementary material for: Cytoglobin regulates NO-dependent cilia motility and organ laterality during development
Source: Nat Commun. 2023 Dec 14;14:8333. doi: 10.1038/s41467-023-43544-0 (PMC10721929; doi:10.1038/s41467-023-43544-0)
Supplement: Supplementary file 3 — Description of Additional Supplementary Files [file 41467_2023_43544_MOESM3_ESM.pdf]

Description of Additional Supplementary Files

Supplementary Movie 1. Left heart looping in wt embryo. Representative video (60 frames/s) of the heart beat in a 2 days post fertilization (dpf) wt embryo. Ventral view.

Supplementary Movie 2. Right heart looping in *$cygb2^{801a}$* mutant. Representative video (60 frames/s) of the heart beat in a 2 dpf *$cygb2^{801a}$* embryo. Ventral view.

Supplementary Movie 3. Beads tracks in wt embryo KV. Representative video (100 frames/s) of fluorescent beads injected into the KV of wt embryos.

Supplementary Movie 4. Beads tracks in *$cygb2^{801a}$* embryo KV. Representative video (100 frames/s) of fluorescent beads injected into the KV of *$cygb2^{801a}$* embryos.

Supplementary Movie 5. Beads tracks in wt embryo KV treated with cPTIO. Representative video (100 frames/s) of fluorescent beads injected into the KV of wt embryos treated with the NO scavenger cPTIO.

Supplementary Movie 6. Beads tracks in *$cygb2^{801a}$* embryo KV treated with DETA/NO. Representative video (100 frames/s) of fluorescent beads injected into the KV of *$cygb2^{801a}$* embryos treated with the NO donor DETA/NO.
