# Supplementary material for: Cytoglobin regulates NO-dependent cilia motility and organ laterality during development
Source: Nat Commun. 2023 Dec 14;14:8333. doi: 10.1038/s41467-023-43544-0 (PMC10721929; doi:10.1038/s41467-023-43544-0)
Supplement: Supplementary file 10 — Reporting Summary [file 41467_2023_43544_MOESM10_ESM.pdf]

nature portfolio

Corresponding author(s): Paola Corti and Mark Gladwin

Last updated by author(s): 11/17/2023

Reporting Summary

Nature Portfolio wishes to improve the reproducibility of the work that we publish. This form provides structure for consistency and transparency in reporting. For further information on Nature Portfolio policies, see our Editorial Policies and the Editorial Policy Checklist.

Please do not complete any field with "not applicable" or n/a. Refer to the help text for what text to use if an item is not relevant to your study.
For final submission: please carefully check your responses for accuracy; you will not be able to make changes later.

Statistics

For all statistical analyses, confirm that the following items are present in the figure legend, table legend, main text, or Methods section.

n/a	Confirmed	
☐	☑	The exact sample size (*n*) for each experimental group/condition, given as a discrete number and unit of measurement
☐	☑	A statement on whether measurements were taken from distinct samples or whether the same sample was measured repeatedly
☐	☑	The statistical test(s) used AND whether they are one- or two-sided<br>*Only common tests should be described solely by name; describe more complex techniques in the Methods section.*
☑	☐	A description of all covariates tested
☐	☑	A description of any assumptions or corrections, such as tests of normality and adjustment for multiple comparisons
☐	☑	A full description of the statistical parameters including central tendency (e.g. means) or other basic estimates (e.g. regression coefficient) AND variation (e.g. standard deviation) or associated estimates of uncertainty (e.g. confidence intervals)
☑	☐	For null hypothesis testing, the test statistic (e.g. *F*, *t*, *r*) with confidence intervals, effect sizes, degrees of freedom and *P* value noted<br>*Give P values as exact values whenever suitable.*
☑	☐	For Bayesian analysis, information on the choice of priors and Markov chain Monte Carlo settings
☑	☐	For hierarchical and complex designs, identification of the appropriate level for tests and full reporting of outcomes
☑	☐	Estimates of effect sizes (e.g. Cohen's *d*, Pearson's *r*), indicating how they were calculated

Our web collection on statistics for biologists contains articles on many of the points above.

Software and code

Policy information about availability of computer code

Data collection	ZEN software, Olympus LS, Image lab software, Quantstudio5 (RTqPCR), Sievers Windows 95/98/NT (version 3.21 PNN)
Data analysis	ImageJ, FiJi, GraphPad Prism 9, Origin 2021

For manuscripts utilizing custom algorithms or software that are central to the research but not yet described in published literature, software must be made available to editors and reviewers. We strongly encourage code deposition in a community repository (e.g. GitHub). See the Nature Portfolio guidelines for submitting code & software for further information.

Data

Policy information about availability of data

All manuscripts must include a data availability statement. This statement should provide the following information, where applicable:
- Accession codes, unique identifiers, or web links for publicly available datasets
- A description of any restrictions on data availability
- For clinical datasets or third party data, please ensure that the statement adheres to our policy

Source data are provided with this paper

Research involving human participants, their data, or biological material

Policy information about studies with human participants or human data. See also policy information about sex, gender (identity/presentation), and sexual orientation and race, ethnicity and racism.

Reporting on sex and gender	not applicable
Reporting on race, ethnicity, or other socially relevant groupings	not applicable
Population characteristics	not applicable
Recruitment	not applicable
Ethics oversight	not applicable

Note that full information on the approval of the study protocol must also be provided in the manuscript.

Field-specific reporting

Please select the one below that is the best fit for your research. If you are not sure, read the appropriate sections before making your selection.

☑ Life sciences ☐ Behavioural & social sciences ☐ Ecological, evolutionary & environmental sciences

For a reference copy of the document with all sections, see nature.com/documents/nr-reporting-summary-flat.pdf

Life sciences study design

All studies must disclose on these points even when the disclosure is negative.

Sample size	For experiments involving quantification of laterality defects we chose a minimum of 50 embryos per experiment and the sample size was determined by the minimum amount of embryos collected from one breeding pair. To overcome the variability due to specific breeding conditions we considered multiple breedings for analysis, averaged the results observed in embryos collected from each breeding and plot these as biological variables. We determined that a minimum n=4 (where each n represents a pool of 50 embryos) was sufficient owing to internal controls (given by the baseline laterality defects observed in wt embryos from multiple breedings). For cilia length experiments, we measured and averaged at least 15 cilia per animal, representing a single data point, given by the minimum number of cilia clearly detectable for each animal. We determine that a minimum n=4 biological replicates was sufficient, owing to controls measured in normal conditions in agreement with values previously shown in the literature.
Data exclusions	Data were not excluded from analysis.
Replication	All replication attempts were successful and observed marker expression pattern were consistent with orthogonal methods and previously known results.
Randomization	Embryos and cilia used for imaging were selected randomly, however we considered for measurements only samples were cilia and heart were clearly detectable by confocal imaging.
Blinding	The analysis of laterality defects was conducted blinded. For the other experiments were blinding was not possible because experimental conditions were evident from the data, quantifications were performed using internal normalization applied equally to all conditions and replicates for a given condition. Thresholds for detecting cilia and laterality defects were chosen for each condition based on objective imaging properties that appeared indistinguishable across conditions.

Behavioural & social sciences study design

All studies must disclose on these points even when the disclosure is negative.

Study description	
Research sample	
Sampling strategy	
Data collection	
Timing	
Data exclusions	
Non-participation	
Randomization	

Ecological, evolutionary & environmental sciences study design

All studies must disclose on these points even when the disclosure is negative.

Study description

Research sample

Sampling strategy

Data collection

Timing and spatial scale

Data exclusions

Reproducibility

Randomization

Blinding

Did the study involve field work? ☐ Yes ☐ No

Field work, collection and transport

Field conditions

Location

Access & import/export

Disturbance

Reporting for specific materials, systems and methods

We require information from authors about some types of materials, experimental systems and methods used in many studies. Here, indicate whether each material, system or method listed is relevant to your study. If you are not sure if a list item applies to your research, read the appropriate section before selecting a response.

Materials & experimental systems

n/a	Involved in the study	
☐	☑	Antibodies
☒	☐	Eukaryotic cell lines
☒	☐	Palaeontology and archaeology
☐	☑	Animals and other organisms
☒	☐	Clinical data
☒	☐	Dual use research of concern
☒	☐	Plants

Methods

n/a	Involved in the study	
☒	☐	ChIP-seq
☒	☐	Flow cytometry
☒	☐	MRI-based neuroimaging

Antibodies

Antibodies used

Anti-Cygb2 polyclonal antibody (Thermo Scientific, Pierce Protein Biology, 1:200 dilution, custom produced in guinea pig), anti-Cygb2113-130 peptide antibody (Thermo Scientific, Pierce Protein Biology, 1:200 dilution, custom produced in rabbit), human anti-Cygb antibody (Sigma HPA017757 1:25 dilution, SantaCruz 66855, 1:500 dilution), anti-beta-actin (A2066 Sigma, 1:200 dilution), anti-acetylated tubulin (Sigma T7451, 1:500 dilution), anti-iNOS (NOVUS NB300, 605ss, 1:200 dilution). Secondary antibodies used were used diluted 1:1000: anti guinea pig AlexaFluor488 (Thermo-Fisher A-11073) anti rabbit AlexaFluor 488 (Thermo-Fisher A-11034), anti mouse AlexaFluor 488 (Thermo-Fisher A-11001), anti rabbit Cy3 (Thermo-Fisher A-10520), anti mouse Cy5 (Thermo-Fisher A-105254), anti-rabbit HRP-conjugated antibody (Abcam ab6789).

Validation

The anti cytoglobin antibodies were validated by using multiple antibodies against the same target and on knockout animals (fish and mouse). Additionally, Sigma HPA017757, A2066, T7451 are anhanced validated antibodies by the manifacturer, NOVUS NB300-605 is validated by biological strategies by the manufacturer.

Eukaryotic cell lines

Policy information about cell lines and Sex and Gender in Research

Cell line source(s)	
Authentication	
Mycoplasma contamination	
Commonly misidentified lines (See ICLAC register)	

Palaeontology and Archaeology

Specimen provenance	
Specimen deposition	
Dating methods	

☐ Tick this box to confirm that the raw and calibrated dates are available in the paper or in Supplementary Information.

Ethics oversight	

Note that full information on the approval of the study protocol must also be provided in the manuscript.

Animals and other research organisms

Policy information about studies involving animals; ARRIVE guidelines recommended for reporting animal research, and Sex and Gender in Research

Laboratory animals	Danio rerio, strains: AB, Tg(fli1:EGFP)y1); Tg (sox17:GFP)s870, and Tg (myl7:DsRed)s879. Only zebrafish embryos were used for this study. Mus musculus, strain C57BL/6. The mice were all 20 months old females.
Wild animals	No wild animals were used in this study.
Reporting on sex	Only zebrafish embryos were used for this study. Adult mice used were all females.
Field-collected samples	No field-collected samples were used in this study.
Ethics oversight	Animals studies were approved by the University of Pittsburgh Institutional Animal Care and Use Committee (Protocol # 21059400 and 21099953), and University of Maryland Institutional Animal Care and Use Committee (Protocol # 1022015).

Note that full information on the approval of the study protocol must also be provided in the manuscript.

Clinical data

Policy information about clinical studies

All manuscripts should comply with the ICMJE guidelines for publication of clinical research and a completed CONSORT checklist must be included with all submissions.

Clinical trial registration	
Study protocol	
Data collection	
Outcomes	

Dual use research of concern

Policy information about dual use research of concern

Hazards

Could the accidental, deliberate or reckless misuse of agents or technologies generated in the work, or the application of information presented in the manuscript, pose a threat to:

No	Yes	
☑	☐	Public health
☑	☐	National security
☑	☐	Crops and/or livestock
☑	☐	Ecosystems
☑	☐	Any other significant area

Experiments of concern

Does the work involve any of these experiments of concern:

No	Yes	
☐	☐	Demonstrate how to render a vaccine ineffective
☐	☐	Confer resistance to therapeutically useful antibiotics or antiviral agents
☐	☐	Enhance the virulence of a pathogen or render a nonpathogen virulent
☐	☐	Increase transmissibility of a pathogen
☐	☐	Alter the host range of a pathogen
☐	☐	Enable evasion of diagnostic/detection modalities
☐	☐	Enable the weaponization of a biological agent or toxin
☐	☐	Any other potentially harmful combination of experiments and agents

Plants

Seed stocks

Novel plant genotypes

Authentication

ChIP-seq

Data deposition

☐ Confirm that both raw and final processed data have been deposited in a public database such as GEO.

☐ Confirm that you have deposited or provided access to graph files (e.g. BED files) for the called peaks.

Data access links
May remain private before publication.

Files in database submission

Genome browser session
(e.g. UCSC)

Methodology

Replicates

Sequencing depth

Antibodies

Peak calling parameters

Data quality

Software

Flow Cytometry

Plots

Confirm that:

- [ ] The axis labels state the marker and fluorochrome used (e.g. CD4-FITC).
- [ ] The axis scales are clearly visible. Include numbers along axes only for bottom left plot of group (a 'group' is an analysis of identical markers).
- [ ] All plots are contour plots with outliers or pseudocolor plots.
- [ ] A numerical value for number of cells or percentage (with statistics) is provided.

Methodology

Sample preparation

Instrument

Software

Cell population abundance

Gating strategy

- [ ] Tick this box to confirm that a figure exemplifying the gating strategy is provided in the Supplementary Information.

Magnetic resonance imaging

Experimental design

Design type

Design specifications

Behavioral performance measures

Imaging type(s)

Field strength

Sequence & imaging parameters

Area of acquisition

Diffusion MRI ☐ Used ☐ Not used

Preprocessing

Preprocessing software

Normalization

Normalization template

Noise and artifact removal

Volume censoring

Statistical modeling & inference

Model type and settings

Effect(s) tested

Specify type of analysis: ☐ Whole brain ☐ ROI-based ☐ Both

Statistic type for inference

(See Eklund et al. 2016)

Correction

Models & analysis

n/a	Involved in the study
☐	☐ Functional and/or effective connectivity
☐	☐ Graph analysis
☐	☐ Multivariate modeling or predictive analysis

Functional and/or effective connectivity

Graph analysis

Multivariate modeling and predictive analysis

This checklist template is licensed under a Creative Commons Attribution 4.0 International License, which permits use, sharing, adaptation, distribution and reproduction in any medium or format, as long as you give appropriate credit to the original author(s) and the source, provide a link to the Creative Commons license, and indicate if changes were made. The images or other third party material in this article are included in the article's Creative Commons license, unless indicated otherwise in a credit line to the material. If material is not included in the article's Creative Commons license and your intended use is not permitted by statutory regulation or exceeds the permitted use, you will need to obtain permission directly from the copyright holder. To view a copy of this license, visit http://creativecommons.org/licenses/by/4.0/
