# Supplementary material for: Cytoglobin regulates NO-dependent cilia motility and organ laterality during development
Source: Nat Commun. 2023 Dec 14;14:8333. doi: 10.1038/s41467-023-43544-0 (PMC10721929; doi:10.1038/s41467-023-43544-0)
Supplement: Supplementary file 11 — Source Data [file 41467_2023_43544_MOESM11_ESM.zip › Rochon Source Data.pdf]

Rochon et al.

Source data are provided with this paper

**Figure 1C**	*wt*	*cygb2* [801a]
	90	61.9
	98	76.9
	85.227273	68.055556
	82.926829	67.701863
	82.926829	45
	82.926829	37.931035
	81.395349	53.571429

**Figure 1D**	*wt*	*cygb2* [801b]
	89.189189	48.913044
	80	70.454546
	100	71.666667
	96.875	55.555556
	78.947368	69.565217
	85.9375	71.014493

**Figure 1F**	Right	Bilateral	Left
wt	1.5	6	92.5
cygb2 [801a]	11	19.3	69.7
wt	0	6.3	93.7
cygb2 [801a]	9.1	15.2	75.7
wt	2.25	0.75	97
cygb2 [801a]	9	19	72
wt	5.9	3.9	90.2
cygb2 [801a]	28.8	0	71.2

Figure 2C

wt	$cygb2^{801a}$
8.35664	4.411731
7.263167	3.6243
7.41205	6.085421
8.128208	3.6409
8.554452	4.198
8.041473	4.653185
6.661737	3.9191
6.1542	4.0049103
6.163857	4.3273731
	3.8727121

Figure 2D

	2 microtubule	1 microtubul	microtubules
wt	36	50	14
$cygb2^{801a}$	17	25	59

Figure 2G

uninjected	***50pg mRNA***	***00pg mRNA***	***uninjected***	***50pg mRNA***	***00pg mRNA***
0.1544859	0.4547889	0.1216313	0.1238847	0.1559086	0.1153654
0.1725772	0.2978259	0.126903	0.093673	0.1381126	0.1394214
0.1675257	0.1665386	0.3774839	0.0864692	0.1461393	0.1079165
0.1675257		0.3182419	0.0001191	0.0823149	0.1079165
0.180997			0.040345	0.0748335	0.2527628
0.0294635			0.0196942		0.1167474
0.1888796			0.0680149		0.1306699
0.0641186			0.1773796		0.384661
0.0660073			0.1547964		0.1157406
0.0995104			0.1809958		0.3571472
0.442048					0.3353066
0.2885232					0.2863631
0.4050379					0.2424003
0.2882691					

Figure 3B

wt	$cygb2^{801a}$
0.942918	0.81932
0.80723	1.022515
1.032661	0.627325
1.907986	1.165345
1.2303965	1.09464
1.0253919	0.5349109
0.8375957	0.8717953
	0.5602459
	0.2153324
	0.3436017
	0.1990459
	0.4054423
	0.6580993

Figure 3F

co-MO	*nos2b-MO*
4.9386875	2.0623333
4.1685882	3.0517222
4.6520909	3.0101539
3.8833	3.106
5.2334546	3.1114546
5.5455	2.7809286
4.9572222	2.382
4.909875	3.3603529
4.65975	3.3045
4.7599444	2.56785

Figure 3G

co-MO	*nos2b-MO*
82.9	41.6
92.6	56.7
95.5	65.3
84.9	63.6
88.8	58.8

Figure 3I

co-MO	*gucy1a-MO*
4.87375	4.82725
4.233	4.01175
5.62625	5.1785
3.90225	4.46825
5.2115	3.847
4.6986667	3.7116667
5.61	3.94775
4.54775	2.46725
5.878625	3.182125
5.879125	3.5125
5.228375	3.221875
5.782375	4.1925
5.29925	3.988375

Figure 3J

co-MO	*gucy1a-MO*
90.3	53.5
90.2	64.2
80.1	57.3
92.5	53.2

Figure 3L

wt	*cygb2* [801a]	*wt*	*cygb2* [801a]
22.14938	14.0448	27.9186	44.79839
12.96842	10.03016	30.40759	45.58838
16.09886	9.473622	25.14485	35.30092
17.13428	21.99734	28.08923	40.55232
28.64847	22.57349	29.85487	36.27153
29.4072	20.82025	34.60273	
31.79441	24.59652		
22.5254	9.23211		
19.4034	15.12111		
19.99021	11.1201		
21.1001			
23.2011			
25.2101			
26.1001			
15.1002			
21.1211			
30.3201			

Figure 4B

DMSO	cPTIO	NaOH/PBS	DETA/NO
14.9237	22.167091	8.1706	9.33775
22.946	8.077	4.3272	8.3905833
10.4947	10.45	5.0833	8.4150833
17.5261	10.052091	6.041	9.3265833
14.2351	10.982	4.2173	10.48125
16.4143	9.1351818	6.3121	14.626667
11.4926	17.180546	4.0084	13.825167
8.1138	5.8304546	5.7969	8.5974167
14.7976	7.8205455	3.4837	18.843
13.5575	16.652636	7.8014	8.5348462
16.3288	16.614	6.8782	18.739308
11.2616	19.967636	3.1222	11.207308
17.1765	19.298364	4.1462	9.9336154
17.8559	12.711455	6.8574	9.1771539
23.7161	7.0942727	4.9674	9.9879231
41.205	12.686909	4.6092	9.2319231
35.5924	5.2491818	3.9458	14.348769
25.9528	7.4242727	3.7722	19.318
27.3411	6.3379091	18.1314	9.9714167
	7.1561818	9.1778	9.54425
	13.556727	14.108	11.563667
	13.257273	22.348	10.48875
	12.722818	15.9307	10.432396
	8.3276364	10.6277	10.6995
	7.318	8.692	8.4038333
	11.568182	10.8443	10.687692
	7.3344546	5.4755	15.738615
		7.3686	12.539231
		9.7927	11.67
		6.278	18.980727
		6.425	16.224546
		9.013	16.684504
		6.708	20.042182
		5.2171	15.207909
		10.8835	13.533636

Figure 4C

DMSO	cPTIO	NaOH/PBS	DETA/NO
89	91	69.230769	97.435897
90	90.5	64	88
85.7	98.04	70.833333	87.5
98		64	88

Figure 4E

	right	bilateral	left
NaOH/PBS	12	5	83
NaOH/PBS	28	27	45
2-4 cells	35	25	40
Oblong	29	13	58
Dome	13	5	82
50%Epib	6	8	86

Figure 4F

DMSO	cinaciguat	DMSO	cinaciguat
94.5	97	79.2	86.1
97.3	96.8	80.1	85.8
98	98.4	78.3	88.2
88.2	88.9	70.2	92.3
		74.3	87.7
		72.7	89.1

Figure 4G

uninjected	*nos2b-mRNA*	uninjected	*nos2b-mRNA*
90	98.7	74	80
86.9	76.67	58.7	73.3
80.5	80	49	67.2
87	82	50	71.1
90	77.6	54.5	61.1
75	75.7	38.7	62.4
		60.2	76.5

Figure 5B

	wt	*ko*
	5.49362805	4.4959326
	5.26303	4.14476
	4.91317	4.76286
	5.13912	4.76286
	5.67012222	4.41562

Figure 5C

wt	*ko*
15.451661	5.8937994
9.1883997	7.4309194
13.710091	3.0219976
22.606304	0.062217

Figure 5F

10 min	+NOS/+CYGB	42.13559	50.16102	46.40678	45.87288	47.52542
	+NOS/-CYGB	28.55932	30.16102	30.05085	29.83898	3.881356
	-NOS/+CYGB	7.279661	5.686441	4.855932	3.889831	
	-NOS/-CYGB	-0.20339	1.152542	-2.40678		
30 min	+NOS/+CYGB	46.28814	51.65254	52.91525	52.72034	49.30508
	+NOS/-CYGB	44.47458	43.77119	43.29661	45.16949	
	-NOS/+CYGB	1.711864	2.491525	4.042373	6.330508	
	-NOS/-CYGB	1.09322	-3.23729	1.186441	-2.4661	

Figure 5E

Time	NADPH only	iNOS -CaM	iNOS + CaM	nos2b + CaM
0	-4.06E-04	-3.11E-05	-3.33E-04	4.58E-04
12	-1.74E-04	0.00149	9.21E-05	0.00211
24	1.09E-04	0.00261	5.96E-04	0.0027
36	-3.47E-04	0.00429	7.87E-04	0.00334
48	2.73E-04	0.00476	0.00167	0.00439
60	3.84E-04	0.0058	0.00238	0.00493
72	5.11E-04	0.00677	0.00185	0.00604
84	6.46E-04	0.00744	0.00246	0.00646
96	9.97E-04	0.00893	0.02001	0.00781
108	8.34E-04	0.00867	0.00362	0.00813
120	0.00148	0.00982	0.00422	0.00921
132	0.00201	0.01081	0.00609	0.00965
144	0.002	0.01174	0.00712	0.01089
156	0.00163	0.01193	0.00763	0.01172
168	0.00175	0.01272	0.00852	0.01208
180	0.00215	0.01381	0.00951	0.01286
192	0.00274	0.01375	0.01052	0.01351
204	0.00305	0.01515	0.01129	0.01351
216	0.0023	0.01526	0.01219	0.01497
228	0.00257	0.01546	0.01247	0.01487

240	0.0031	0.01669	0.01355	0.01542
252	0.00309	0.01664	0.01413	0.01586
264	0.00307	0.01711	0.01463	0.01661
276	0.00308	0.01743	0.01498	0.01759
288	0.0036	0.01799	0.01551	0.018
300	0.00334	0.01825	0.01625	0.01864
312	0.00353	0.01868	0.01681	0.01834
324	0.00396	0.01856	0.01743	0.01907
336	0.00418	0.01901	0.01741	0.01942
348	0.00398	0.01945	0.01832	0.01981
360	0.00434	0.01942	0.01836	0.02004
372	0.00386	0.02047	0.01893	0.02049
384	0.00403	0.02048	0.01895	0.02074
396	0.00493	0.02046	0.01922	0.02104
408	0.00469	0.02088	0.01961	0.02138
420	0.00522	0.02109	0.02016	0.02121
432	0.00453	0.02133	0.02016	0.02159
444	0.0049	0.02124	0.02042	0.02219
456	0.00554	0.02192	0.02108	0.02301
468	0.00521	0.02197	0.02131	0.02255
480	0.00562	0.02217	0.02127	0.02267
492	0.00577	0.02236	0.02167	0.02345
504	0.00541	0.02243	0.02233	0.02315
516	0.00581	0.02286	0.02208	0.02345
528	0.00613	0.02262	0.02229	0.02327
540	0.00588	0.02348	0.02288	0.02389
552	0.00603	0.02332	0.02228	0.02408
564	0.00607	0.02352	0.02316	0.02455
576	0.00649	0.02346	0.02333	0.02441
588	0.00607	0.02373	0.02336	0.02458
600	0.00633	0.02396	0.02412	0.02426
612	0.00685	0.02363	0.02356	0.02457
624	0.00671	0.02396	0.02366	0.02487
636	0.00641	0.0239	0.02404	0.02475
648	0.00674	0.02445	0.02436	0.0253
660	0.00666	0.02376	0.02451	0.02514
672	0.0069	0.02411	0.02443	0.02492
684	0.00734	0.02427	0.02438	0.02504
696	0.00773	0.0243	0.02436	0.02514
708	0.00715	0.02441	0.02475	0.02558
720	0.0075	0.02506	0.02505	0.0252
732	0.00736	0.02455	0.02524	0.02554
744	0.00715	0.0244	0.02508	0.02524

756	0.0079	0.02457	0.02473	0.02548
768	0.0073	0.02455	0.02485	0.02575
780	0.00803	0.02501	0.02513	0.02603
792	0.00781	0.02514	0.02523	0.02599
804	0.00811	0.025	0.02516	0.02634
816	0.00784	0.02536	0.02547	0.026
828	0.00793	0.02517	0.02529	0.02614
840	0.00828	0.02531	0.02503	0.02651
852	0.00809	0.02544	0.02577	0.02652
864	0.00813	0.02539	0.02594	0.02654
876	0.00765	0.02546	0.02602	0.02624
888	0.00848	0.02547	0.02583	0.02594
900	0.00875	0.02581	0.02535	--
912	0.00829	0.02583	0.02596	--
924	0.00827	0.02597	0.02608	--
936	0.00878	0.0255	0.02619	--
948	0.00897	0.02625	0.02618	--
960	0.0086	0.026	0.02607	--
972	0.00908	0.026	0.02597	--
984	0.00903	0.02579	0.02641	--
996	0.00883	0.02594	0.02604	--
1008	0.00895	0.02636	0.02615	--
1020	0.00991	0.02601	--	--
1032	0.00928	0.02618	--	--
1044	0.00868	0.02643	--	--
1056	0.00911	0.02616	--	--
1068	0.00943	0.02644	--	--
1080	0.00896	0.02595	--	--
1092	0.00934	0.02596	--	--
1104	0.00938	0.02598	--	--
1116	0.00941	0.02619	--	--
1128	0.01003	0.02646	--	--
1140	0.00909	0.02654	--	--
1152	0.00981	0.02638	--	--
1164	0.00951	0.02663	--	--
1176	0.01031	--	--	--
1188	0.00956		--	--
1200	0.00968		--	--
1212	0.0102		--	--
1224	0.00985		--	--
1236	0.0102		--	--
1248	0.01016		--	--
1260	0.00964		--	--

1272	0.01014	--	--
1284	0.00995	--	--
1296	0.01009	--	--
1308	0.01023	--	--
1320	0.01026	--	--
1332	0.01018	--	--
1344	0.01038	--	--
1356	0.01032	--	--
1368	0.01005	--	--
1380	0.01023	--	--
1392	0.01054	--	--
1404	0.01062	--	--
1416	0.01035	--	--
1428	0.01071	--	--
1440	0.01078	--	--
1452	0.01078	--	--
1464	0.01065	--	--
1476	0.01059	--	--
1488	0.01074	--	
1500	0.0106		
1512			
1524			
1536			
1548			

Supp Fig.S1B

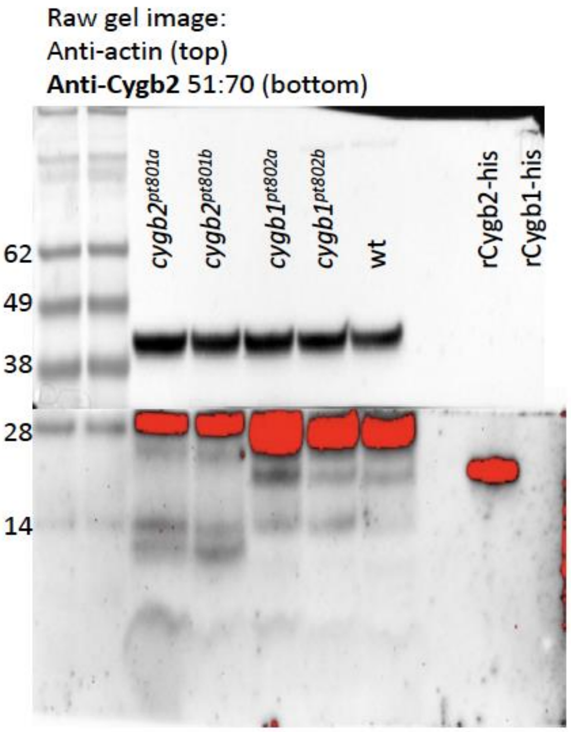

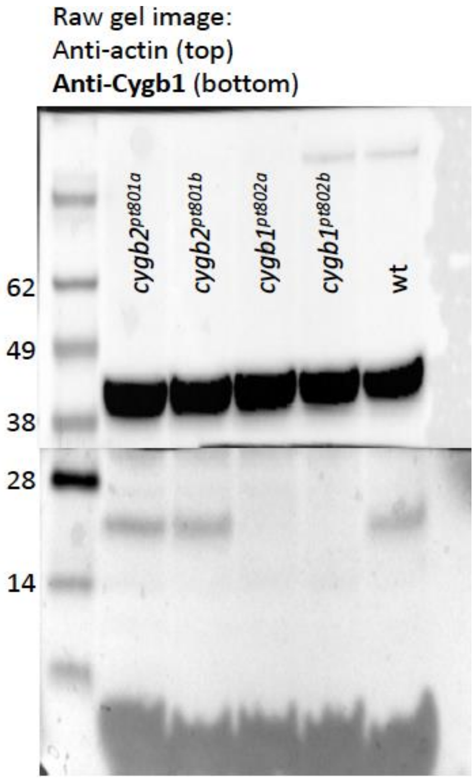

quantification	wt	*cygb2* *801a*	*cygb2* *801b*
	1.3242843	0.0946492	0.4293535
	0.6972977	0.2605651	0.3045865
	1.4571621	0.2468297	0.401822
	1.0840971		0.3744065
	0.7827732		
	0.9621822		
	1.1126419		

Supp Fig.S1C

	Right	Bilateral	Left
wt	8	3	89
cygb2^{801a}	29	3	68
wt	12	8	80
cygb2^{801b}	32	19	49

Supp Fig.S1D

wt	*cygb2* *801a*
3.16	2.7
3.1	3.1
3.18	3.02
3.12	2.92

3	2.8
3.24	3.04
3.22	2.94
3.2	2.68
	2.92
	2.66
	2.5
	2.86
	3
	2.68
	3.08
	2.68
	3.06
	3.04
	3.1
	3.08

Supp Fig.S1E

wt	*cygb2* ^{801a}
3.8	3.3
4.1	3.5
3.7	3.2
3.8	3.5

Supp Fig.S1F

	curved	straight
wt	0	100
cygb2^{801a}	24	76

Supp Fig.S1I

	Right	Bilateral	Left
wt	0	5	95
cygb1^{802}	3	4	93

Supp Fig.S2B

wt	$cygb2^{pt801b}$
5.4411667	5.3341667
6.283	2.110875
4.2793333	2.2405
5.122375	2.4215
5.820125	3.4311667
	2.5295
	3.8545
	3.273

Supp Fig.S2C

wt	$cygb2^{801a}$
13000	15000
13000	11000
16000	10000
11000	11000
10000	14000
9000	13000
9000	12000
12000	13000
10000	13000
10000	15000
12000	12000
10000	15000
16000	10000
15000	11000
11000	11000
12000	14000
16000	13000
14000	12000

Supp Fig.S2E

6 som		8 som	
wt	$cygb2^{801a}$	*wt*	$cygb2^{801a}$
163.925	211.532	274.103	249.33
133.886	144.977	241.996	243.143
174.161	157.226		253.679
180.15	125.809		
144.274	149.334		
171.034	185.083		
177.652	182.138		
192.189	181.158		
141.733	156.647		
175.672			

12 som		14 som	
wt	$cygb2^{801a}$	*wt*	$cygb2^{801a}$
152.841	142.153	78.55	156.703
157.154	128.338	101.903	59.731
173.665	236.968	128.062	62.01
216.152	215.127	95.945	75.989
181.935	205.36	146.311	173.381

179.674	243.943	50.439	154.727
107.467	172.027	48.048	99.01
112.342	189.884	0	155.524
161.756	161.794	77.713	130.989
	167.677	62.35	105.928

Supp Fig.S3C

	co-MO	*cygb2-MO*
	8.35664	4.62068
	7.263167	5.226391
	7.41205	3.285019
	8.128208	3.610343
	8.554452	3.802917
	8.041473	4.3805
	6.661737	4.9004
	6.1542	4.19645
	6.163857	3.9835
		3.658308

Supp Fig.S3D

	co-MO	*cygb2-MO*	*cygb2* ^{801a}
	19	12	10
	19	20	29
	10	20	26
	14	14	33
	50	13	26
	24	50	10
	20	46	19
	24	53	10
	31	35	12
		36	27

Supp Fig.S3F

	Right	Bilateral	Left
co-MO	0	23	76
cygb2-MO	33	42	25

Supp Fig.S4A		Right	Bilateral	Left
	co-MO	17	1	82
	nos2b- MO	50	9	42
	co- MO	28	9	63
	nos2b- MO	41	3	57

Supp Fig.S4B		Right	Bilateral	Left
	uninjected	3	2	95
	nos2b-mRNA	1	1	99
	nos2bMO	29	7	65
	nos2b-mRNA/nos2bMO	14	3	84

		Right	Bilateral	Left
Supp Fig.S4C	Co-MO	8	2	90
	2.5 ng	10	6	84
	5 ng	24.7	21.8	53.5

		Right	Bilateral	Left
Supp Fig.S4D	Co-MO	8	0	92
	1 ng	20.5	8	71.5
	2 ng	29	18	53

Supp Fig.S5C

	right	bilateral	left
Sod. Ph		2	98
SOD+Catalase	3.4	8.6	87.9
Sod. Ph	11.6	11.5	76.9
SOD+Catalase	27.6	6.1	66.3

Supp Fig.S5D

	right	bilateral	left
DMSO	5.2	8.6	86.2
SODm 20uM	8.1	12.9	79
SODm 200uM	9.4	18.9	71.7
DMSO	35.3	15.7	49
SODm200nM	33	7.7	59.3
SODm2uM	40.8	11.1	48.1
SODm20uM	33.3	12.4	54.3
SODm 200uM	40	10	50

Supp Fig.S5A

Time (s)	0 nM SOD			7 nM SOD	14 nM SOD	35 nM SOD
0	0	0	*0**	0	0	0
6	0.00849346	0.007802	*0.00472635*	0.004138	0.002861	0.0001907
12	0.01613614	0.0150628	*0.00921619*	0.0077601	0.005134	0.0002903
18	0.02508313	0.0236813	*0.01539523*	0.0121988	0.0063	0.0003813
24	0.03112073	0.0303236	*0.01938287*	0.0134729	0.006867	0.0036268
30	0.03860528	0.0404588	*0.02457792*	0.0181205	0.01106	0.006643
36	0.04269852	0.0480032	*0.02987317*	0.020292	0.013222	0.0067924
42	0.05280565	0.0550219	*0.03502995*	0.0239385	0.015215	0.0108966
48	0.05891762	0.0612154	*0.04083351*	0.028393	0.015593	0.0133538
54	0.06364134	0.0715584	*0.04669175*	0.0315169	0.017916	0.0141906
60	0.07086888	0.078287	*0.04950458*	0.0330594	0.022407	0.0145544
66	0.07738086	0.0877034	*0.05431906*	0.04169	0.022591	0.019069
72	0.08699192	0.0941282	*0.05906582*	0.0406665	0.022743	0.0157301
78	0.09464624	0.1008422	*0.06368283*	0.0452197	0.025588	0.0169011
84	0.10131928	0.1107956	*0.066564**	0.0493416	0.027052	0.0190997
90	0.10709795	0.1157336	*0.0722768**	0.0494613	0.030116	0.0201044
96	0.11424116	0.1246525	*0.07926658*	0.0514717	0.031584	0.0224303

102	0.12029218	0.1320029	*0.0836705*	0.0550083	0.033852	0.0254462
108	0.12768453	0.1384585		0.0586927	0.035499	0.0257128
114	0.13275459	0.14632		0.0607472	0.040586	0.0249807
120	0.14034986	0.1531467		0.0631633	0.039872	0.0289946
126	0.14886227	0.1609907		0.069752	0.041502	0.028453
132	0.15653128	0.1686678		0.0693654	0.044005	0.0312843
138	0.16156296	0.174114		0.0753439	0.044776	0.0335838
144	0.17004091	0.1808197		0.0758037	0.046979	0.034087
150	0.17367831	0.1863414		0.0779	0.048556	0.0372084
156	0.17918395	0.1923623		0.0799201	0.050072	0.0384954
162	0.18774857	0.2000813		0.0826928	0.052075	0.0387662
168	0.19592471	0.2071357		0.084259	0.054727	0.0428673
174	0.20041054	0.2128312			0.05674	0.0420812

Supp Fig.S5E Cygb1

25 nM zfCyg	100 nM zfCygb	500 nM zfCy	1000 nM zfCygb1
0	0	0	0
0.0092039	0.00316039	0.0074772	0.005378
0.0173677	0.00837487	0.0123479	0.014025
0.0259558	0.01523501	0.0184174	0.020082
0.0336812	0.02277842	0.0288749	0.026037
0.0430837	0.02923152	0.0353858	0.031723
0.0518644	0.03720096	0.0407666	0.039986
0.0613691	0.04497659	0.0465638	0.045642
0.0702055	0.0499467	0.0549727	0.050313
0.0783687	0.05825064	0.0587231	0.056825
0.0901017	0.06432995	0.0675117	0.062869
0.0997318	0.06932193	0.0752631	0.066904
0.1043488	0.07504469	0.0800298	0.074904
0.1085992	0.08219874	0.0870648	0.080678
0.1184512	0.08923537	0.0939482	0.087748
0.1256393	0.0941942	0.1004088	0.094758
0.1328018	0.10181335	0.1060013	0.097354
0.1407932	0.10932511	0.1131286	0.101171
0.1488629	0.11248371	0.1200384	0.107133
0.15702	0.12107739	0.126283	0.111821
0.1652118	0.12583166	0.134384	0.11727
0.1733023	0.13266826	0.1391755	0.122411
0.1816489	0.14053384	0.1460314	0.127286
0.1886525	0.14575854	0.1536947	0.134478
0.1963205	0.15137491	0.1576416	0.13687
0.2044392	0.15819031	0.1616054	0.14405
0.21204	0.16693804	0.1678324	0.147682

0.2178559	0.16976655	0.1755209	0.153543
0.2276774	0.17689633	0.1804068	0.157914
0.2336914	0.18155414	0.1858995	0.16408

Supp Fig.S5B Cygb2

25 nM zfCyg	100 nM zfCygb	500 nM zfCy	1000 nM zfCygb2
0	0	0	0
0.0101435	0.0050828	0.0091499	0.012252
0.0188699	0.01241008	0.0175471	0.015494
0.0280742	0.02019316	0.0271627	0.02334
0.0357904	0.02754292	0.0382377	0.031777
0.0458601	0.04049619	0.0505821	0.039463
0.0544105	0.04541628	0.0557974	0.048167
0.063629	0.05260663	0.063593	0.057364
0.0700243	0.06165274	0.0724492	0.063279
0.0781984	0.0681165	0.082225	0.070091
0.08708	0.07693802	0.0876655	0.080493
0.0964779	0.08579983	0.097125	0.088383
0.1031041	0.09468325	0.1059995	0.095723
0.111617	0.10345669	0.1159162	0.103854
0.1202317	0.09411447	0.1247319	0.112377
0.1280476	0.11712976	0.133322	0.119303
0.134727	0.12476351	0.1401937	0.126282
0.1430906	0.13330506	0.151938	0.134132
0.1490991	0.14390044	0.158602	0.142066
0.1576177	0.14971982	0.167955	0.151091
0.1678399	0.1574591	0.1738359	0.158128
0.174972	0.16486497	0.1830966	0.165016
0.180799	0.17248278	0.1909415	0.173392
0.1873966	0.17798357	0.1978479	0.180625
0.196267	0.18751402	0.2060275	0.188965
0.2060784	0.19709118	0.2168346	0.196051
0.2128754	0.20289718	0.2238183	0.201597
0.2214054	0.20920466	0.2320265	0.209668
0.2282936	0.2182454	0.2400119	0.218437
0.2355328	0.22454156	0.247137	0.225087

Supp Fig.S5F

100 nM CYGB
0
0.006622
0.019287
0.027188
0.037817
0.046485
0.056104
0.066041
0.076662
0.084601
0.094351
0.10262
0.111501
0.121619
0.129167
0.139071
0.148674
0.157016
0.165096
0.173734

Supp Fig.S6A

right	bilateral	left	
NaOH/PBS	1.6949153	0	98.305085
NaOH/PBS	5.7692308	25	69.230769
125 µM DET	6.17	11.1	82.72
250 µM DET	0	2.5641026	97.435897
500 µM DET	7.6923077	12.820513	79.48718

Supp Fig.S6B

wt	cygb2^{801a}	wt	cygb2^{801a}	wt	cygb2^{801a}
6.4444	4.797	4.3159	4.3887	5.6425	4.1354
4.7393	3.8637	5.1618	4.778	4.698	3.6088
5.4511	3.9502	3.8906	4.4901	3.8868	3.3205714
5.9587	4.4431	5.0122	4.3284	4.93	4.5517
4.5927	3.3935	4.6963	4.1237	5.0288	4.3599
4.7246		4.71	4.1679	5.3945	4.5746667
5.3697		5.0983	3.8656	5.2731	
4.599		4.9341	3.4792	4.8622	
		4.3985	4.871		
		4.8947	3.9551		
		4.8677	4.9329		

Supp Fig.S6C

	right	bilateral	left
DMSO	5.9	5.9	88.2
80uM cinaci	7.4	3.7	88.9
DMSO	18.4	9.2	72.4
20uM cinaci	15.6	9.6	74.8
40uM cinaci	8	5.3	86.7
80uM cinaci	6.4	3.9	89.7

Supp Fig.S6D

	right	bilateral	left
uninjected	12.35	7.45	80.2
nos2b- mRN	18.27	5.06	76.67
uninjected	27.7	15.6	56.7
nos2b-mRN	14.995	6.53	78.475

Supp Fig.S7A

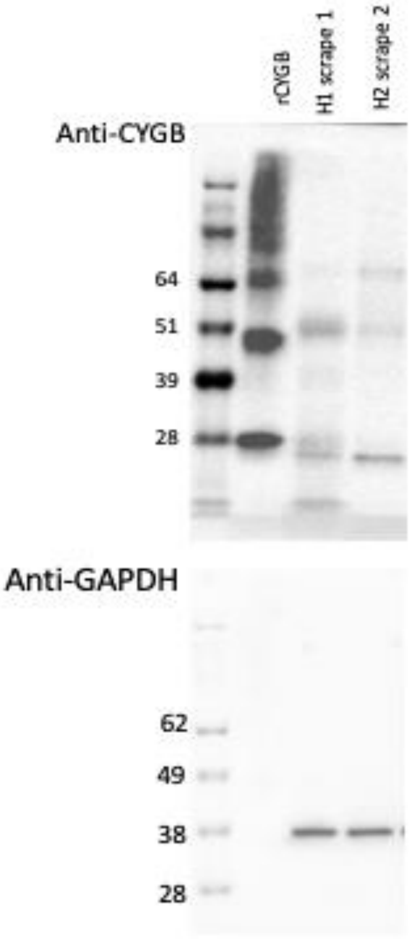

Supp Fig.S7B

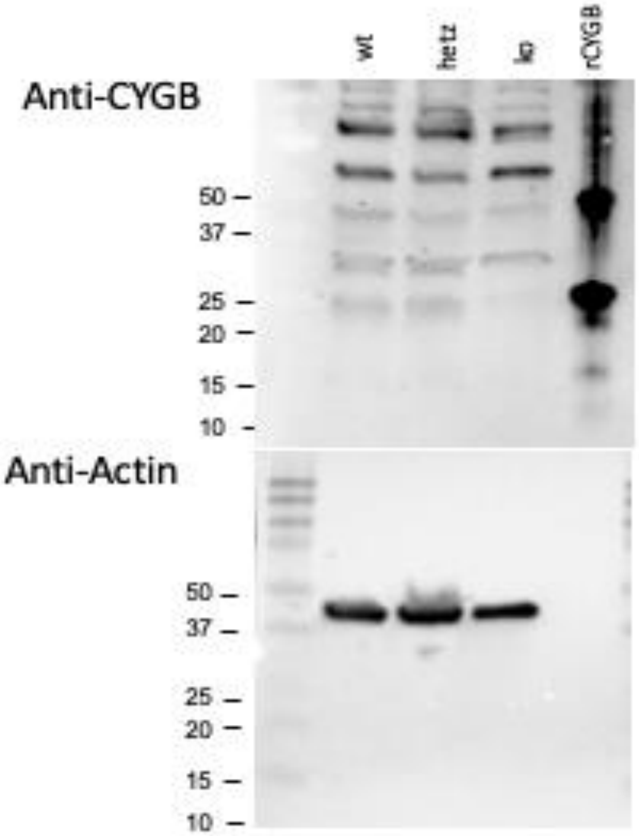

Supp Fig.S7D	*wt*	*ko*
eNOS	1.229736	1.576858
	0.480536	1.166245
	1.570339	1.147052
	1.091023	1.104057
iNOS	0.947134	1.279457
	0.521616	0.696883
	1.644711	0.944622
	1.247993	1.152703

Supp Fig.S7E

wt	*ko*
100	132.05492
110.61873	106.00109
89.381272	133.39901

Supp Fig.S7F

wt	*ko*
107.0463	54.88313
92.953705	96.453143
103.0173	71.550896
93.965398	45.902102
